# Complete Genome Sequences of Two Human Oral Microbiome Commensals: *Streptococcus salivarius* ATCC 25975 and *S. salivarius* ATCC 27945

**DOI:** 10.1128/genomeA.00536-17

**Published:** 2017-06-15

**Authors:** Robert R. Butler, Jahna T. A. Soomer-James, Michel Frenette, Jean-François Pombert

**Affiliations:** a Department of Biology, Illinois Institute of Technology, Chicago, Illinois, USA; b Département de Biochimie, Microbiologie et Bio-informatique, Faculté des Sciences et de Génie, Université Laval, Québec, Québec, Canada

## Abstract

*Streptococcus salivarius* strains are significant contributors to the human oral microbiome. Some possess unique fimbriae that give them the ability to coaggregate and colonize particular oral structures. We present here the complete genomes of *Streptococcus salivarius* Lancefield K^−^/K^+^ strains ATCC 25975 and ATCC 27945, which can and cannot, respectively, produce fimbriae.

## GENOME ANNOUNCEMENT

*Streptococcus salivarius* is a well-known commensal in the human oral microbiome. A variety of strains inhabit various subregions of the oral cavity (1) and presumably adapt to these niches. Certain *S. salivarius* strains are classified as Lancefield K^−^ because they lack the K antigen (2). These strains have also been described as having fimbriae in a uniform distribution across their outer surface, differentiating them from K^+^ strains, which possess various fibrillar appendages (3). Fimbriae confer adherence properties, allowing for bacterial coaggregation with other members of the oral microbiota, such as other streptococci or periodontopathogens, and adherence to specific oral mucosa (4–6). Here, we present the complete circular genomes of two *S. salivarius* strains, ATCC 25975 (originally deposited as *S. salivarius* Andrewes and Horder) and ATCC 27945 (originally deposited as *S. hominis*), which are Lancefield K^−^ and K^+^, respectively.

Pure cultures of the two strains were provided by American Type Culture Collection (ATCC). Cultures of both strains were grown shaking at 37°C overnight in tryptone glucose yeast extract medium. Genomic DNA was extracted using the MasterPure Gram-positive (Epicentre, Madison, WI, USA) DNA purification kit with the addition of 2 units of mutanolysin per mL of culture. Sequencing was conducted on a PacBio RS II sequencer (Pacific Biosciences, Menlo Park, CA, USA) using two single-molecule real-time (SMRT) cells per strain and P5-C3 chemistry (one SMRT cell of ATCC 25975 was run using P6-C4 chemistry) at the University of Michigan DNA Sequencing Core (Ann Arbor, MI, USA). DNA was purified using AMPure XP beads (Beckman Coulter, Inc., Brea, CA, USA) and sheared to between 10 and 20 kb (*N*_50_: ATCC 25975, 13,296 bp; ATCC 27945, 11,893 bp) with g-TUBEs (Covaris, Woburn, MA, USA).

Genomes were assembled using the HGAP2 protocol in SMRT-Analysis version 2.3.0 (7). The assembled contigs were trimmed to circularization and then corrected via sequential runs of the RS_Resequencing protocol (one run for the ATCC 25975 chromosome, three runs for the ATCC 25975 plasmid, and two runs for the ATCC 27945 chromosome). The ATCC 25975 assembly contained both a complete chromosome (2,199,793 bp, 39.9% G+C, 275.4× coverage) and a previously unidentified plasmid (126,555 bp, 35.9% G+C, 230.8× coverage). The ATCC 27945 assembly contained only the complete chromosome (2,108,274 bp, 40.2% G+C, 449.5× coverage).

The genomes were annotated using the NCBI Prokaryotic Genome Annotation Pipeline version 3.1 (8). A total of 2,044/1,847 coding sequences, 68/68 tRNAs, 18/18 rRNAs, 4/4 ncRNAs, and 1/0 CRISPR array were annotated in the ATCC 25975/27945 genomes, respectively.

### Accession number(s).

These complete genome projects were deposited in GenBank under the accession numbers CP015283.1 (ATCC 25975 chromosome), CP015284.1 (ATCC 25975 plasmid), and CP015282.1 (ATCC 27945 chromosome).
